# In Vitro Evaluation of Whole Blood Hemostatic Function at a Level 1 Trauma Center

**DOI:** 10.7759/cureus.98468

**Published:** 2025-12-04

**Authors:** Brian R Czarkowski, Rigel Hall, Andrea C Vazquez-Loreto, Jody L Handschug, Kristina M Kupanoff, Dih-Dih Huang, Michael D Jones, Hahn Soe-Lin, James N Bogert, Jordan A Weinberg

**Affiliations:** 1 Department of Trauma/Acute and General Surgery, St. Joseph's Hospital and Medical Center, Phoenix, USA; 2 Department of Trauma/Acute and General Surgery, Creighton University School of Medicine, St. Joseph's Hospital and Medical Center, Phoenix, USA; 3 Department of Surgery, University of Arizona College of Medicine - Phoenix, Phoenix, USA

**Keywords:** cold-stored whole blood, fresh frozen plasma transfusion, hemostasis, platelet transfusion, reconstituted whole blood, thromboelastography (teg)

## Abstract

Background: Cold-stored whole blood (CSWB) is increasingly used in civilian trauma care, but concerns remain regarding its hemostatic efficacy, particularly platelet function. This study aimed to evaluate the thromboelastogram (TEG) profiles of emergency-release whole blood units available in the blood bank of our Level 1 trauma center. We hypothesized that CSWB would show a storage age-dependent decline in platelet function and that its hemostatic profile would resemble that of reconstituted whole blood (RWB).

Methods: TEG 6S analyses were performed on samples from 10 CSWB and 10 RWB units. Parameters assessed included reaction time (R time), functional fibrinogen (FF), and maximum amplitude (MA). Results were compared between groups and analyzed relative to storage age.

Results: R times were within normal limits for all samples. For FF, 2/10 CSWB samples were below normal compared to none of the RWB samples (P = 0.474). For MA, 7/10 CSWB samples were below normal compared to none of the RWB samples (P = 0.003). All RWB MA values exceeded those of CSWB. No correlation was found between CSWB MA values and storage age.

Conclusion: In vitro platelet function, as assessed by TEG 6S, was below normal in most CSWB units but normal in all RWB samples. These findings suggest that transfusing hemostatically deficient CSWB may be suboptimal in managing trauma-related coagulopathy.

## Introduction

The majority of preventable deaths from traumatic injuries are due to hemorrhagic shock and occur within the first 24 hours of injury [1,2]. Renewed interest in whole blood resuscitation has emerged since the wars in Iraq and Afghanistan, leading to its increasing use in civilian trauma centers. Since the 1970s, hemorrhagic shock has been managed with component therapy, packed red blood cells (PRBCs), platelets, and fresh frozen plasma (FFP), and, since the 2010s, in a 1:1:1 ratio [3,4]. More recent studies have suggested a survival benefit with whole blood resuscitation [5-8], while others have reported similar mortality outcomes compared with component therapy [9-11].

Only fresh, warm whole blood has the physiologic advantage of being fully hemostatic, with preserved platelet function and clotting factor activity. Zielinski et al. demonstrated that the in vitro hemostatic properties of fresh whole blood showed rotational thromboelastography (ROTEM) parameters well within the normal range [12]. Fresh whole blood is collected via on-demand donation (the “walking blood bank” used by the military) and must be transfused within 72 hours to maintain its coagulation properties [13]. However, its use is not authorized in civilian practice, where only cold-stored whole blood (CSWB) is available.

Previous studies examining the effects of cold storage on the hemostatic properties of whole blood have shown variable degrees of deterioration over time, typically following a linear trend [12,14-20]. Many of these studies used leukoreduced whole blood, though not all, and it appears that leukoreduction may exacerbate hemostatic decline during storage [16,20].

The purpose of this study was to compare the coagulation profiles of CSWB and reconstituted whole blood (RWB; PRBC-to-FFP-to-platelets in a 1:1:1 ratio) using thromboelastography (TEG) on units stocked in our blood bank. We aimed to determine whether CSWB demonstrates in vitro hemostatic inferiority compared with RWB.

## Materials and methods

This study was conducted at St. Joseph’s Hospital and Medical Center, a Level 1 trauma center in Phoenix, Arizona. Blood specimens were collected by the hospital blood bank without any personal identifying information. This study did not involve human research participants and was therefore not subject to institutional review board oversight.

Blood product samples were taken from blood units stored in the hospital blood bank for clinical use. Products included leukoreduced CSWB preserved with citrate-phosphate-dextrose (CPD), leukoreduced RBCs preserved with AS-3 (Nutricel), FFP, and leukoreduced apheresis platelets. Leukoreduction was performed by the blood supplier using a platelet-sparing filter shortly after collection (prestorage).

Samples were obtained from 10 unique CSWB units and 30 unique component units (10 RBCs, 10 platelets, and 10 FFPs) from products on hand for patient transfusion. For CSWB, 5 mL samples were withdrawn from refrigerated units. To create RWB for comparison, 2.25 mL RBCs, 2.25 mL FFP, and 0.375 mL platelets were combined, yielding 10 unique RWB samples. Plasma was thawed for three hours before reconstitution, and RBCs were taken directly from refrigeration. All platelet samples used for RWB were from seven-day-old platelets stored at room temperature.

Following sample preparation and equilibration to 37°C, TEG was performed on all 20 samples by a medical laboratory scientist using the TEG 6s device (Haemonetics Corp., Boston, MA, USA). Each sample was assigned a storage age in days, based on the age of the CSWB unit or the RBC component in the RWB samples. Manufacturer reference ranges were used to define normal values: CK-R 4.6-9.1 minutes, CRT-MA 52-69 mm, and CFF-MA 15-32 mm [21]. The minimum CRT-MA value reported by the TEG test is 40 mm; values < 40 mm were recorded as 40 mm for statistical analysis.

The proportions of assays outside the normal range were compared using Fisher’s exact tests and reported with Pearson chi-square test statistics. Mean values between groups were compared using independent-samples t-tests. Statistical analyses were performed using IBM SPSS Statistics for Windows, Version 27.0 (IBM Corp., Armonk, NY, USA). A p-value <0.05 was considered statistically significant.

## Results

TEG 6s parameters for the 10 RWB and 10 CSWB samples were compared. The storage age of each sample, along with its corresponding TEG 6s parameters (CK-R, CRT-MA, and CFF-MA), is presented in Table 1. The CSWB samples ranged in age from 6 to 21 days, while the RWB samples ranged from 7 to 35 days.

**Table 1:** Whole and reconstituted blood parameters

Blood group	Unit	Age (days)	CK-R (min)	CRT-MA (mm)	CFF-MA (mm)
Whole	1	6	5.7	55	26.5
	2	8	6.6	42.4	13.1
	3	8	7.2	<40.0	16.6
	4	12	5.9	49.9	16.3
	5	12	6.7	52	19.8
	6	14	8.5	<40.0	16.3
	7	14	6.4	42.6	14.2
	8	20	6.5	53.5	19.2
	9	20	8.1	45.7	17.9
	10	21	6.8	41.7	24.6
Reconstituted	11	7	5.9	63.4	21.9
	12	9	6.2	62.9	22.3
	13	12	5.7	62.1	21.7
	14	16	5.7	62	21.6
	15	17	5.8	62.5	21.3
	16	20	6	62.6	22.3
	17	24	5.8	62.3	21.3
	18	26	6.5	63	20.8
	19	31	6.3	62.5	21.7
	20	35	6.5	62.4	21.3

**Figure 1:**
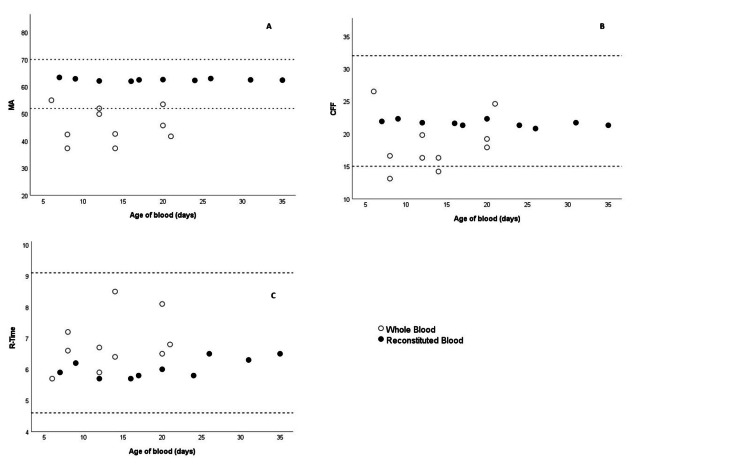
Scatterplot between thromboelastography parameters and age of blood

The number of TEG results for each group falling outside the defined normal ranges is presented in Table 2. No abnormal values were observed for any TEG parameter among the RWB samples. For the CSWB samples, all CK-R values were within the normal range. However, 7/10 CRT-MA values from CSWB samples were abnormal, which was significantly higher compared to the RWB group (70% vs. 0%, P = 0.003). Two of the CFF-MA values in the CSWB group were abnormal, compared with none in the RWB group (20% vs. 0%, P = 0.474). There was no statistically significant difference in storage age between the two groups (RWB: 19.7 ± 9.3 days vs. CSWB: 13.5 ± 5.4 days, P = 0.085). Storage age was not correlated with any of the three TEG parameters: CRT-MA r(18) = 0.340, P = 0.143; CFF-MA r(18) = 0.198, P = 0.404; CK-R r(18) = 0.063, P = 0.791 (Figure 1A-1C).

**Table 2:** Comparison of blood parameters between cold-stored whole blood and reconstituted whole blood Fisher’s exact test was used to compare proportions, and independent samples t-tests were used to compare mean values.

		CK-R	CRT-MA	CFF-MA
Values outside of the normal range				
Whole blood		0 (0%)	7 (70%)	2 (20%)
Reconstituted		0 (0%)	0 (0%)	0 (0%)
Chi-square test-statistic (degrees of freedom)		NA	10.8 (1)	2.2(1)
P-value		NA	0.003	0.474
Mean values				
Whole blood		6.8 ± 0.9	45.7 ± 6.5	18.5 ± 4.2
Reconstituted		6.0 ± 0.3	62.6 ± 0.4	21.6 ± 0.5
T-statistic (degrees of freedom)		-2.7 (18)	8.1 (18)	2.3 (18)
P-value		0.02	<0.001	0.044

Mean values for the three TEG assays are compared in Table 2. In the RWB group, the mean CK-R values were significantly lower than those in the CSWB group (P = 0.020), while the mean CRT-MA (P < 0.001) and CFF-MA (P = 0.044) values were significantly higher.

## Discussion

There are several advantages to CSWB for hemorrhagic shock resuscitation, including the logistical benefit of providing erythrocytes, platelets, and coagulation factors within a single product. In prehospital or austere settings, CSWB may offer major benefits since the storage requirements for platelets and plasma often limit their availability. In Level 1 trauma centers, however, reconstituting whole blood by transfusing components in a 1:1:1 ratio has become standard practice, allowing for early and simultaneous administration of PRBC, prethawed or liquid plasma, and platelets. Previous studies evaluating the effects of cold storage on the hemostatic properties of CSWB have shown variable but generally progressive deterioration, particularly in platelet function, over time [12,14-20]. Having recently adopted CSWB use in our trauma center, we sought to compare its hemostatic properties, measured by TEG 6s, with those of RWB prepared from standard components. We found that most CSWB units demonstrated abnormal TEG 6s profiles, while all RWB samples were normal. 

To our knowledge, this is the first study comparing CSWB and RWB using the TEG 6s system. Limitations of the older TEG 5000 include manual pipetting requirements and susceptibility to vibration artifacts. The newer TEG 6s platform was designed to address these issues and has been shown to be reliable in trauma settings [22]. Nonetheless, it is possible that the early platelet dysfunction we observed, reflected by reduced MA, may relate in part to TEG 6s assay characteristics. 

The significant hemostatic impairment in the CSWB group, particularly the low MA values, reflects platelet dysfunction. Prior studies using TEG 5000 have demonstrated a linear decline in MA with storage time [14,18,23], and similar findings have been reported with ROTEM [12,15,17,19]. Notably, all CSWB units in this study were leukoreduced using a platelet-sparing filter, as is standard practice in the USA. The effect of leukoreduction on platelet function, even with platelet-sparing filters, remains unclear. Haddaway et al. found that although platelet counts in leukoreduced CSWB declined over time, MA remained within the normal range [23]. In our study, platelet counts were not measured, but variations in platelet count among units may have contributed to the MA variability observed with TEG 6s. Assen et al. also demonstrated decreasing platelet counts and MA in non-leukoreduced CSWB over time, while both Remy et al. and Siletz et al. reported worse hemostatic function in leukoreduced compared to non-leukoreduced CSWB [14,16,20]. Thus, despite the use of platelet-sparing filters, leukoreduction may adversely affect platelet function. Since most blood suppliers in the USA provide only leukoreduced CSWB, it is important to recognize that platelet function may be inconsistent or suboptimal in these units.

When comparing TEG 6s parameters between CSWB and RWB, R time, representing coagulation factor activity, was slightly longer in CSWB, though still within normal limits. Although heat-labile factors V and VIII degrade over time in CSWB, this did not appear to affect clot initiation, consistent with the findings of Assen et al. [14].

This study has several limitations. It is a single-institution study with a small sample size, and in vitro findings may not directly reflect clinical performance. Only a single TEG tracing was performed for each sample to preserve blood units for clinical use. Unlike studies using dedicated research specimens, we sampled “off-the-shelf” products intended for transfusion, which allowed continued use of these units afterward. Other laboratory assays such as cell counts were not performed for the same reason. However, these products were subject to standard quality control measures. Finally, although in vitro TEG abnormalities may not precisely predict in vivo hemostatic efficacy, it is reasonable to infer that products demonstrating abnormal TEG profiles may provide less effective hemostasis in clinical practice.

## Conclusions

In this study, we demonstrated that the in vitro hemostatic characteristics of CSWB are significantly inferior to those of RWB prepared using the standard 1:1:1 component ratio. The most notable difference was in platelet function, as reflected by the TEG 6s maximum amplitude parameter. To our knowledge, this is the first study to compare CSWB and RWB using the TEG 6s system. The in vivo impact of transfusing CSWB units with abnormal TEG profiles remains unclear. Nonetheless, our findings suggest that trauma centers should critically reconsider the use of leukoreduced CSWB for hemorrhage management in trauma patients. Although leukoreduction is common practice and often mandated, it may impair platelet function in CSWB and warrants reevaluation. Finally, regional trauma systems should assess the optimal allocation of this limited resource; CSWB may be best reserved for prehospital environments and critical access hospitals that traditionally have access only to emergency PRBC transfusions.
